# Association of skeletal muscle relaxers and antihistamines on mortality, hospitalizations, and emergency department visits in elderly patients: a nationwide retrospective cohort study

**DOI:** 10.1186/1471-2318-15-2

**Published:** 2015-01-27

**Authors:** Carlos A Alvarez, Eric M Mortensen, Una E Makris, Dan R Berlowitz, Laurel A Copeland, Chester B Good, Megan E Amuan, Mary Jo V Pugh

**Affiliations:** Pharmacy Practice Department, Texas Tech University Health Sciences Center, Forest Park Rd, Dallas, TX USA; Clinical Science Department, University of Texas Southwestern, Harry Hines, Dallas, TX USA; Internal Medicine Department, University of Texas Southwestern, Harry Hines, Dallas, TX USA; Departments of Medicine and Health Policy and Management, Boston University, Springs Rd, Bedford, MA USA; Center for Healthcare Organization and Implementation Research, Springs Rd, Bedford, MA USA; Center for Applied Health Research – Health Outcomes Core, Central Texas Veterans Health Care System and Baylor Scott & White Health, Birdcreek Dr, Temple, TX USA; Departments of Medicine and Pharmacy, University of Pittsburgh, University Dr C, Pittsburgh, PA USA; South Texas Veterans Healthcare System and the Department of Epidemiology and Biostatistics at the University of TX Health Science Center, San Antonio, TX USA

**Keywords:** Aged, Antihistamines, Skeletal muscle relaxant, Adverse drug events, Healthcare effectiveness data and information set, Mortality, Hospitalizations, Emergency service

## Abstract

**Background:**

High-risk medication exposure in the elderly is common and associated with increased mortality, hospitalizations, and emergency department (ED) visits. Skeletal muscle relaxants and antihistamines are high-risk medications commonly prescribed in elderly patients. The objective of this study was to determine the association between skeletal muscle relaxants or antihistamines and mortality, hospitalizations, and emergency department visits.

**Methods:**

This study used a new-user, retrospective cohort design using national Veteran Affairs (VA) data from 128 hospitals. Veterans ≥65 years of age on October 1, 2005 who received VA inpatient/outpatient care at least once in each of fiscal year (FY) 2005 and FY 2006 were included. Exposure to skeletal muscle relaxants and antihistamines was defined by the National Committee for Quality Assurance Healthcare Effectiveness Data and Information Set measures for high-risk medications in the elderly. Primary outcomes identified within one year of exposure were death, ED visit, or hospitalization; ED visits or hospitalizations due to falls and fracture were also assessed. Propensity score matching (1 to 1 match) was used to balance covariates between exposed patients and non-exposed patients.

**Results:**

In this cohort of 1,807,404 patients 55,566 patients were included in the propensity-matched cohort for skeletal muscle relaxants and 60,058 patients were included in the propensity-matched cohort for anti-histamines. Mortality was lower in skeletal muscle relaxants-exposed patients (adjusted odds ratio [AOR] 0.87, 95% CI 0.81-0.94), but risk of emergency care (AOR 2.25, 95% CI 2.16-2.33) and hospitalization (AOR 1.56, 95% CI 1.48-1.65) was higher for patients prescribed skeletal muscle relaxants. Similar findings were observed for emergency and hospital care for falls or fractures. Mortality (AOR 1.93, 95% CI 1.82-2.04), ED visits (AOR 2.35, 95% CI 2.27-2.43), and hospitalizations (AOR 2.21, 95% CI 2.11-2.32) were higher in the antihistamine-exposed group, with similar findings for falls and fractures outcomes.

**Conclusion:**

Skeletal muscle relaxants and antihistamines are associated with an increased risk of ED visits and hospitalizations in elderly patients. Antihistamines were also associated with an increased risk of death, further validating the classification of these drug classes as “high risk”.

## Background

A recent population-based study determined that high-risk medication exposure in the elderly is common and associated with an increase in mortality, hospitalizations, and emergency department (ED) visits [1]. Prevention of drug related problems and inappropriate prescribing in the elderly have been identified as a priority by the Institute of Medicine [2]. To address these problems explicit quality measures have been developed by experts to identify medications whose risks outweigh potential benefits [3–6]. The National Committee on Quality Assurance (NCQA) developed a Healthcare Effectiveness Data and Information Set (HEDIS) quality measure to examine use of High Risk Medications in the Elderly (HRME) [6].

The most common HRME drugs prescribed in the aforementioned study were skeletal muscle relaxants and first-generation antihistamines [1]. These drugs are potentially dangerous in the elderly due to their side effects that cause sedation, confusion, and extremity weakness, all factors associated with falls [3–6]. However, evidence regarding the clinical impact in prescribing antihistamines and skeletal muscle relaxants in older patients are lacking [7, 8].

Therefore, we conducted a retrospective cohort study to determine whether these commonly prescribed medications are associated with increased death, ED visits, and hospitalizations in elderly patients after adjusting for potential confounders and selection bias using propensity-score matching. Based on prior studies we hypothesized that individuals prescribed skeletal muscle relaxants and antihistamines would be more likely to have poor clinical outcomes when compared to elderly patients not exposed to these agents.

## Methods

This study was approved by the Institutional Review Boards at the University of Texas Health Science Center at San Antonio, South Texas Veterans Health Care System, and the Bedford Veterans Affairs (VA) medical center. Relevant permissions were obtained to access patient data.

### Study design, patients, and setting

This study was a new-user, retrospective cohort design using national VA administrative data. Veterans were included if they were ≥65 years of age on October 1, 2005 and received VA inpatient/outpatient care at least once in each of fiscal year 2005 (FY 2005) and FY 2006 (October 1, 2004 through September 30, 2006). Patients were excluded if they were in a VA nursing home or transitional care unit >60 days in FY 2005 or FY 2006, exposed to skeletal muscle relaxants or antihistamines prior to FY 2006, or had a subsequent new skeletal muscle relaxant/antihistamine exposure in FY 2007. Each patient was used only once as either a control or drug exposed subject.

### Data sources

We used inpatient and outpatient demographic, utilization, and comorbidity data from the VA Medical SAS Datasets. Pharmacy data were extracted from the Pharmacy Benefits Management (PBM) dataset. Prescription data in the VA PBM included prescription start date, generic drug name, and day supply for each drug dispensed. Mortality information was obtained from the VA Vital Status file, which has a sensitivity of ~98% for veterans’ deaths [9]. Unique patient identifiers linked information across these databases.

### Exposure definition

Patients exposed to skeletal muscle relaxants or antihistamines were identified using the generic drug name in the VA PBM dataset. Exposure to these agents was defined by the NCQA HEDIS measures for high risk medications in the elderly [6]. Antihistamines included diphenhydramine, hydroxyzine, promethazine, cyproheptadine, chlorpheniramine, or tripelennamine. Skeletal muscle relaxants included methocarbamol, cyclobenzaprine, carisoprodol, chlorzoxazone, metaxalone, or orphenadrine. New users of skeletal muscle relaxants or antihistamines were defined as those with no exposure in FY 2005 who were subsequently exposed in FY 2006. The index date was classified as the day of the first new prescription. Those without exposure (comparator arm) had an index date of October 1, 2005.

### Outcomes

Our primary outcomes were death, ED visit, or hospitalization within one year of the index date. The date of death was identified using the VA Vital Status files, emergency care was documented using the outpatient VA stop code pair 102–101 prior to 03/01/2007 or 130 (emergency care), 131 (urgent care) after that date, and VA hospital admission and date of admission were identified using inpatient data. ED visits or hospitalizations due to falls and fracture within one year of index date were also assessed using International Classification of Diseases, Ninth Revision (ICD-9-CM) codes E880.X-E888.X, 820.X, and 733.14. Patients were identified using an algorithm developed and tested by NCQA.

### Covariates

Covariates were selected a priori on the basis of clinical relevance and previous research [1, 10, 11].

#### Demographics

Demographic characteristics (age, sex, race/ ethnicity) were identified using data from FY 2004-FY 2006. With the exception of race/ethnicity, these characteristics were well documented and complete in the medical record. Missing demographic data are common in VA files; however, several years of data help allay this problem. Poverty was defined as patients with income under the VA poverty limit and identified by VA means testing. This limit is geographically-adjusted and in 2010 was approximately $29,402.

#### Clinical characteristics

Clinical characteristics included measures that are associated with high disease burden. Higher disease burden, as defined by more medications, more physical comorbidities and psychiatric conditions, place patients at higher risk for adverse outcomes [2, 12]. We also counted the number of unique medications received during FY 2005, including medications prescribed “as needed”. Non-VA medications were not included in this count. ICD-9-CM codes found in VA inpatient and outpatient data (FY 2004- FY2005) were used to identify individuals with physical and psychiatric conditions using the Selim comorbidity indices [13, 14]. These indices were developed to measure chronic disease burden in research involving Veterans. The numbers of chronic disease states from 30 possible conditions (chronic kidney disease is included in this count, but not dementia or falls/fractures) were counted to determine the Selim Physical index. The Selim Psychiatric index includes a count of 6 mental health conditions: schizophrenia, bipolar, depression, substance use disorder, anxiety and post-traumatic stress disorder.

Prior healthcare utilization that indicated disease burden was assessed. Previous studies indicate that older Veterans who receive geriatric care have higher disease burden and increased risk of HRME exposure; therefore, individuals who received geriatric care (outpatient or inpatient) in FY 2005 were identified [11]. Individuals were identified who had one or more encounters of emergency care or hospital admission in FY 2005 as indicators of disease burden. The number of primary care visits in FY 2005 was also counted as an indicator of disease burden and health care utilization [15]. In order to facilitate interpretation of findings we created categorical variables for specific continuous measures. Medications were classified as 0–5, 6–8, 9–11 and 12 or more based on the empirical distribution from prior research [1]. Physical conditions were classified as zero to one, two to three, four to five and six or more chronic conditions; psychiatric conditions as zero, one, and two or more conditions. Based on the empirical distribution, the number of primary care visits was classified as having zero to one, two to four, and five or more visits.

### Statistical analyses

We first provided bivariate analysis comparing those exposed and unexposed in each medication class. In order to reduce confounding by indication we then used propensity score matching to balance measured covariates between patients exposed to antihistamines or skeletal muscle relaxants and unexposed patients [16]. Separate logistic regression models were used to create the propensity score for each drug group, which modeled the probability of antihistamine or skeletal muscle relaxant use given all other study covariates. A routine described by Leuven and Sianesi was utilized to perform nearest-number matching with a caliper of 0.0001 [17]. Adjusted odds ratios (AOR) for the primary and secondary outcomes were then determined using conditional logistic regression models.

Statistical significance was defined as a two-tailed p value ≤ 0.05. All analyses were conducted with STATA 12 (College Station, TX).

## Results

### Baseline characteristics

There were a total of 1,807,404 elderly veteran patients who met inclusion criteria. The patient sample was mostly Caucasian (66%) males (98.4%) with ≥2 physical comorbidities (71.6%) and taking ≥5 medications (51.4%; Tables 1 and 2). Within 1 year of cohort entry, 81,003 patients (4.6%) died, 87,118 patients (4.9%) had one or more hospital admissions, 244,106 patients (13.5%) received emergency care; 6,871 patients (0.4%) visited the emergency department for a fall, and 1,692 (0.09%) patients were hospitalized due to a fall. The composite primary outcome of death, ED visit or hospitalization within 1-year of cohort entry occurred in 320,513 patients (17.7%).

**Table 1:** **Patient characteristics-skeletal muscle relaxant**

Variable N(%)	Unmatched characteristics			Matched Demographic Characteristics (1:1 propensity-scored matching)		
	Incident Muscle Relaxant Use	No Muscle Relaxant Use	P value	Incident Muscle Relaxant Use	No Muscle Relaxant Use	P value
	n = 27786	n = 1779618		n = 27783	n = 27783	
Age Groups						
65-74	16216 (58.4%)	803689 (45.2%)	<0.001	16213 (58.4%)	16205 (58.3%)	0.994
75-84	10207 (36.7%)	818252 (46%)		10207 (36.7%)	10210 (36.8%)	
85+	1363 (4.9%)	157677 (8.9%)		1363 (4.9%)	1368 (4.9%)	
Gender (female)	668 (2.4%)	28458 (1.6%)	<0.001	665 (2.4%)	637 (2.3%)	0.432
Race/Ethnicity						
White	19072 (68.6%)	1173582 (66%)	<0.001	19072 (68.7%)	19083 (68.7%)	1.00
Black	2841 (10.2%)	110042 (6.2%)		2839 (10.2%)	2838 (10.2%)	
Hispanic	1854 (6.7%)	54433 (3.1%)		1853 (6.7%)	1854 (6.7%)	
Other	393 (1.4%)	22436 (1.3%)		393 (1.4%)	391 (1.4%)	
Unknown/missing	3626 (13.1%)	419125 (23.6%)		3626 (13.1%)	3617 (13%)	
Poverty Status						
Under the poverty limit	20873 (75.1%)	1038801 (58.4%)	<0.001	20870 (75.1%)	20886 (75.2%)	0.895
Number of Medications						
0-5	7900 (28.4%)	871000 (48.9%)	<0.001	7900 (28.4)	7900 (28.4)	1.00
6-8	6811 (24.5%)	459144 (25.8%)		6810 (24.5%)	6808 (24.5%)	
9-11	5601 (20.2%)	249228 (14%)		5601 (20.2%)	5595 (20.1%)	
>12	7474 (26.9%)	200246 (11.3%)		7472 (26.9%)	7480 (26.9%)	
Number of Physical Comorbidities						
0-1	5107 (18.4%)	508737 (28.6%)	<0.001	5107 (18.4%)	5123 (18.4%)	0.996
2-3	12041 (43.3%)	844548 (47.5%)		12040 (43.3%)	12040 (43.3%)	
4-5	7387 (26.6%)	329098 (18.5%)		7385 (26.6%)	7385 (26.6%)	
6+	3251 (11.7%)	97235 (5.5%)		3251 (11.7%)	3235 (11.7%)	
Number of Psychiatric Comorbidities						
0	22267 (80.1%)	1555665 (87.4%)	<0.001	22267 (80.1%)	22263 (80.1%)	0.993
1	4093 (14.7%)	179695 (10.1%)		4091 (14.7%)	4099 (14.7%)	
2+	1426 (5.1%)	44258 (2.5%)		1425 (5.1%)	1421 (5.1%)	
ED visits in 2005	8439 (30.4%)	243864 (13.7%)	<0.001	8436 (30.4%)	8441 (30.4%)	0.963
Hospital admissions in 2005	3050 (11%)	96276 (5.4%)	<0.001	3049 (11%)	3046 (11%)	0.968
Geriatric visits in 2005	553 (2%)	42550 (2.4%)	<0.001	553 (2%)	551 (2%)	0.952
Primary Care visits						
0-1	3639 (13.1%)	478963 (26.9%)	<0.001	3639 (13.1%)	3639 (13.1%)	0.993
2-4	14736 (53%)	991503 (55.7%)		14736 (53%)	14749 (53.1%)	
5+	9411 (33.9%)	309152 (17.4%)		9411 (33.9%)	9398 (33.8%)	
**Outcomes**						
Death within 1 year	1414 (5.1%)	79589 (4.5%)	<0.001	1414 (5.1%)	1613 (5.8%)	<0.001
Emergency department visit within 1 year	11169 (40.2%)	232937 (13.1%)	<0.001	11169 (40.2%)	6399 (23.0%)	<0.001
Hospitalization within 1 year	3613 (13%)	83505 (4.7%)	<0.001	3613 (13%)	2430 (8.8%)	<0.001
Emergency department visit for fall within 1 year	384 (1.4%)	6487 (0.4%)	<0.001	384 (1.4%)	176 (0.63%)	<0.001
Hospitalization for fall within 1 year	67 (0.2%)	1625 (0.1%)	<0.001	67 (0.24%)	46 (0.17%)	0.048
Death emergency department visit or hospitalization within 1 year (composite)	12365 (44.5%)	308148 (17.3%)	<0.001	12356 (44.5%)	7821 (28.2%)	<0.001

**Table 2:** **Patient characteristics-antihistamines**

Variable N(%)	Unmatched Characteristics			Matched Demographic Characteristics (1:1 propensity-scored matching)		
Variable N(%)	Incident Antihistamine Use	No Antihistamine Use	P value	Incident Antihistamine Use	No Antihistamine Use	P value
	n = 30031	n = 1777373		n = 30029	n = 30029	
Age Groups						
65-74	14853 (49.5%)	805052 (45.3%)	<0.001	14851 (49.5%)	14848 (49.5%)	0.993
75-84	12728 (42.4%)	815731 (45.9%)		12728 (42.4%)	12738 (42.4%)	
85+	2450 (8.2%)	156590 (8.8%)		2450 (8.1%)	2443 (8.1%)	
Gender (female)	740 (2.5%)	28386 (1.6%)	<0.001	738 (2.5%)	722 (2.4%)	0.672
Race/Ethnicity						
White	21392 (71.2%)	1171262 (65.9%)	<0.001	21392 (71.2%)	21408 (71.3%)	0.995
Black	3197 (10.7%)	109686 (6.2%)		3195 (10.6%)	3196 (10.6%)	
Hispanic	1677 (5.6%)	54610 (3.1%)		1677 (5.6%)	1655 (5.5%)	
Other	482 (1.6%)	22347 (1.3%)		482 (1.6%)	476 (1.6%)	
Unknown/missing	3283 (10.9%)	419468 (23.6%)		3283 (10.9%)	3294 (11%)	
Poverty Status						
Under the poverty limit	23780 (79.2%)	1035894 (58.3%)	<0.001	23778 (79.2%)	23790 (79.2%)	0.602
Number of Medications						
0-5	7451 (24.8%)	871449 (49%)	<0.001	7451 (24.8%)	7447(24.8%)	1.000
6-8	7045 (23.5%)	458910 (25.8%)		7045 (23.5%)	7049 (23.5%)	
9-11	6292 (21%)	248537 (14%)		6292 (21%)	6301 (21%)	
>12	9243 (30.8%)	198477 (11.2%)		9241 (30.8%)	9232 (30.7%)	
Number of Physical Comorbidities						
0-1	5174 (17.2%)	844104 (47.5%)	<0.001	5174 (17.2%)	5160 (17.2%)	0.997
2-3	12485 (41.6%)	328371 (18.5%)		12485 (41.6%)	12497 (41.6%)	
4-5	8114 (27%)	96228 (5.4%)		8113 (27%)	8127 (27.1%)	
6+	4258 (14.2%)	508670 (28.6%		4257 (14.2%)	4245 (14.1%)	
Number of Psychiatric Comorbidities						
0	23260 (77.5%)	1554672 (87.5%)	<0.001	23260 (77.5%)	23263 (77.5%)	0.958
1	4906 (16.3%)	178882 (10.1%)		4906 (16.3%)	4919 (16.4%)	
2+	1865 (6.2%)	43819 (2.4%)		1863 (6.2%)	1847 (6.2%)	
ED visits in 2005	10345 (34.5%)	241958 (13.6%)	<0.001	10343 (34.4%)	10343 (34.4%)	1.000
Hospital admissions in 2005	4472 (14.9%)	94854 (5.3%)	<0.001	4471 (14.9%)	4456 (14.8%)	0.863
Geriatric visits in 2005	867 (2.9%)	42236 (2.4%)	<0.001	867 (2.9%)	847 (2.8%)	0.624
Primary Care visits						
0-1	3975 (13.2%)	478627 (26.9%)	<0.001	3975 (13.2%)	3969 (13.2%)	0.997
2-4	15017 (50%)	991222 (55.8%)		15016 (50%)	15018 (50%)	
5+	11039 (36.8%)	307524 (17.3%)		11038 (36.8%)	11042 (36.8%)	
**Outcomes**						
Death within 1 year	3685 (12.2%)	77318 (4.4%)	<0.001	3684 (12.3%)	2033 (6.8%)	<0.001
Emergency department visit within 1 year	13267 (44.2%)	230839 (13%)	<0.001	13266 (44.2%)	7562 (25.2%)	<0.001
Hospitalization within 1 year	5729 (19.1%)	81389 (4.6%)	<0.001	5729 (19.1%)	2895 (9.6%)	<0.001
Emergency department visit for fall within 1 year	383 (1.3%)	6488 (0.4%)	<0.001	383 (1.3%)	216 (0.7%)	<0.001
Hospitalization for fall within 1 year	109 (0.4%)	1583 (0.1%)	<0.001	109 (0.4%)	56 (0.2%)	<0.001
Death emergency department visit or hospitalization within 1 year (composite)	16142 (53.8%)	304362 (17.1%)	<0.001	16141 (53.8%)	9276 (30.9%)	<0.001

### Skeletal muscle relaxant exposure

A total of 27,786 (1.5%) patients had a new skeletal muscle relaxant exposure in FY 2006. The unadjusted and adjusted odds ratios for the primary and secondary outcomes for the total cohort are provided in Table 3.

**Table 3:** **Association of skeletal muscle relaxant exposure with outcomes**

Variable	Unadjusted OR (95% CI)	Adjusted OR (95% CI)
Death	1.14 (1.09-1.21)	0.87 (0.81-0.94)
ED at one year	4.46 (4.36-4.57)	2.25 (2.16-2.33)
Hospitalization at one year	3.04 (2.93-3.15)	1.56 (1.48-1.65)
ED Falls and Fractures	3.83 (3.45-4.25)	2.20 (1.84-2.63)
Hospitalization Falls and Fractures	2.64 (2.07-3.38)	1.46 (1.001-2.12)
Any outcome (composite)	3.82 (3.73-3.92)	2.04 (1.97-2.12)

From the 1,807,404 elderly veterans identified, a total of 55,566 patients were included in the propensity-matched cohort; 27,783 skeletal muscle relaxant exposed and 27,783 matched unexposed. Balance between key covariates after propensity matching is shown on Table 1. There were no statistically significant differences in measured characteristics between skeletal muscle relaxant exposed patients and those not treated.

Table 3 shows the results from the propensity-matched cohort for the primary and secondary outcomes. The primary composite outcome of death, ED visits, and hospitalizations at one year was significantly higher (AOR 2.04, 95% Confidence Interval [CI] 1.97-2.12) in patients exposed to skeletal muscle relaxants vs. patients not exposed. Mortality at one-year was significantly lower in patients exposed to skeletal muscle relaxants (AOR 0.87, 95% CI 0.81-0.94). ED visits (AOR 2.25, 95% CI 2.16-2.33) and hospitalization (AOR 1.56, 95% CI 1.48-1.65) at one year was significantly higher for patients prescribed skeletal muscle relaxants. ED visits (AOR 2.20, 95% CI 1.84-2.63) and hospitalizations (AOR 1.46, 95% CI 1.001-2.12) for falls or fractures at one year were also significantly higher in patients taking skeletal muscle relaxants.

### Antihistamine exposure

There were 30,031 (1.7%) patients exposed to antihistamines in FY 2006. Table 4 shows the unadjusted odds ratios for the primary and secondary endpoints for all patients included in the study.

**Table 4:** **Association of antihistamine exposure with outcomes**

Variable	Unadjusted OR (95% CI)	Adjusted OR (95% CI)
Death	3.08 (2.97-3.19)	1.93 (1.82-2.04)
ED at one year	5.30 (5.18-5.43)	2.35 (2.27-2.43)
Hospitalization at one year	4.91 (4.77-5.06)	2.21 (2.11-2.32)
ED Falls and Fractures	3.53 (3.18-3.91)	1.78 (1.51-2.11)
Hospitalization Falls and Fractures	4.09 (3.37-4.96)	1.95 (1.41-2.69)
Any outcome (composite)	5.62 (5.50-5.76)	2.60 (2.51-2.69)

For the propensity-matched cohort, we included a total of 60,058 patients; 30,029 patients exposed to antihistamines and 30,029 unexposed. Table 2 shows the balance among key covariates between antihistamine-exposed patients and unexposed after propensity matching. After propensity matching there were no statistically significant differences between the two groups.

Results for the propensity-matched cohort for the primary and secondary outcomes are displayed on Table 4. The primary composite outcome at one year was significantly higher in patients exposed to antihistamines (AOR 2.60, 95% CI 2.51-2.69). Mortality (AOR 1.93, 95% CI 1.82-2.04), ED visits (AOR 2.35, 95% CI 2.27-2.43), and hospitalizations (AOR 2.21, 95% CI 2.11-2.32) at one year were significantly higher in the antihistamine-exposed group. Fall and fracture related ED visits (AOR 1.78, 95% CI 1.51-2.11) and hospitalizations (AOR 1.95, 95% CI 1.41-2.69) were also significantly higher at one year in the antihistamine-exposed group.

## Discussion

Our study demonstrated that exposure to either skeletal muscle relaxants or antihistamines in the elderly were associated with a greater than two-fold increase in ED visits, and/or hospitalizations. Antihistamines were associated with a 93% increase in mortality. Furthermore, our study found that exposure to either of these agents increased fall and/or fracture related ED visits and hospitalizations.

Interestingly, exposure to skeletal muscle relaxants was associated with a 13% reduction in death. We believe this finding may be explained by unmeasured confounding due to the “healthy worker” bias [18]. As an example, physicians use information such as functional status that is not readily measured in the medical record or captured in administrative claims data when prescribing potentially dangerous medications. Although speculative, it is possible that the elderly patients prescribed skeletal muscle relaxants were healthier than those not exposed in unmeasured ways. Moreover, these healthier, more active patients may have experienced more musculoskeletal injuries that would require long-term care or closer monitoring by the clinician. Chronic diseases such as hypertension or diabetes may have been better controlled under these more closely monitored conditions leading to reduced mortality.

Despite the fact that these medications are listed as inappropriate to use in elderly patients, we found the incidence of antihistamine exposure to be 1.7% and skeletal muscle relaxant exposure to be 1.5%. The incidence of antihistamine and skeletal muscle relaxant exposure in this study was similar to reports in the literature [1, 11, 19, 20]. Hospitalization and ED visit rates found in this study are also similar to findings in previous studies [1, 11]. Medications on the NCQA HEDIS HRME list have rarely been shown to be associated with ED visits via adverse drug events (ADE) [12, 21]. Most ADEs that elevate the risk of ED visits in the elderly were derived from a narrow list of commonly used medications such as antithrombotic and anti-diabetic drugs [12]. Although ADEs due to skeletal muscle relaxants or antihistamines may potentially explain some of the increased risk of ED visits or hospitalizations in our study, elderly patients who received prescriptions for these medications may have other underlying poorly controlled chronic conditions that lead to poor outcomes such as hospitalizations and ED visits. HRME has been associated with increased mortality [1]; however, this study, to our knowledge, is the first to examine mortality specifically for antihistamines and skeletal muscle relaxants.

Several studies have validated explicit measures of inappropriate prescribing in the elderly; however, few have examined the clinical impact of specific high-risk medications such as antihistamines and skeletal muscle relaxants [1, 4, 5, 22–24]. In the 2012 update of the Beers Criteria, the consensus expert panel strongly recommended the avoidance of first-generation antihistamines and skeletal muscle relaxants based on only moderate quality of evidence with the exception of hydroxyzine and promethazine, which had high quality evidence. This study provides further support for harmful effects of antihistamines and skeletal muscle relaxants in elderly patients. This is important considering 90% of health plans in the United States use NCQA HEDIS measures to determine provider and health system performance [25]. These measures take into consideration that pharmacokinetics and pharmacodynamics of drugs are altered in elderly patients. Diphenhydramine, a commonly used antihistamine, undergoes substantial first-pass metabolism and is 80-85% protein bound [26, 27]. Elderly patients on medications that are metabolized by the liver, like most antihistamines and skeletal muscle relaxants, may experience more ADEs due to a reduction of up to 40% in hepatic volume and blood flow [28, 29]. Drugs that are highly protein bound, such as diphenhydramine, may also result in more ADEs due to a decrease in serum albumin by 15-20% in elderly patients [28, 29].

Our study has important limitations. First, variables not collected for this study may lead to unmeasured confounding. Our study did not include data, such as functional status, that are not available in the VA datasets. Propensity score matching was performed to balance covariates and control for important clinical comorbidities that are risk factors for mortality, hospitalizations, ED visits, falls, and fractures. Second, over-the-counter status of many antihistamines may have led to an underestimation or misclassification of exposure. Moreover, other antihistamines (e.g., doxylamine and scopolamine) and skeletal muscle relaxants (e.g., baclofen and tizanidine) not listed as HEDIS HRME may have been used by both exposed and non-exposed patients. A non-differential misclassification of antihistamine and skeletal muscle relaxant exposure produces a bias towards the null, potentially making our interpretation of increased risk of events more conservative [30]. Third, the analysis did not account for exposure time or time to event. Skeletal muscle relaxants and antihistamines can be used “as needed” for symptoms rather than scheduled daily like many chronic medications listed as HEDIS HRME. Moreover, this study determined events within one year from exposure, which may strengthen the association as compared to choosing a more distal time-point. Fourth, we did not collect data on drug specific outcome associations. It is unknown whether a specific drug within either class predominately contributed to the observed association. However, the hypothesis was to determine if drug exposure as defined by NCQA HEDIS HRME was associated with outcomes and may not be powered to determine drug-specific associations. Additional research is needed to determine drug-specific associations. Finally, the sample in the study was representative of a VA population and had a preponderance of male patients. Therefore, generalizability to elderly female, non-VA patients is not clear [31].

## Conclusion

This study demonstrates that skeletal muscle relaxants and antihistamines, as listed in the NCQA HEDIS HRME, are associated with adverse events in elderly patients, adding further evidence for the validity of the classification of “high-risk” for these drug classes. Further interventions need to be developed to identify patients at high risk for events and reduce exposure to these medications while still providing acceptable substitutes for patients.

## Abbreviations

ED: Emergency department
VA: Veterans affairs
FY: Fiscal year
AOR: Adjusted odds ratio
CI: Confidence interval
NCQA: National committee on quality assurance
HEDIS: Healthcare effectiveness data and information set
HRME: High risk medications in the elderly
PBM: Pharmacy benefit management
ICD-9-CM: International classification of diseases, ninth revision
OR: Odds ratio
ADE: Adverse drug events.
